# Access To Essential Maternal Health Interventions and Human Rights Violations among Vulnerable Communities in Eastern Burma

**DOI:** 10.1371/journal.pmed.0050242

**Published:** 2008-12-23

**Authors:** Luke C Mullany, Catherine I Lee, Lin Yone, Palae Paw, Eh Kalu Shwe Oo, Cynthia Maung, Thomas J Lee, Chris Beyrer

**Affiliations:** 1 Center for Public Health and Human Rights, Bloomberg School of Public Health, Baltimore, Maryland, United States of America; 2 Global Health Access Program, Mae Sot, Thailand; 3 Burma Medical Association, Mae Sot, Thailand; 4 Karen Department of Health and Welfare, Mae Sot, Thailand; 5 Mae Tao Clinic, Mae Sot, Thailand; National Institute of Child Health and Human Development, United States of America

## Abstract

**Background:**

Health indicators are poor and human rights violations are widespread in eastern Burma. Reproductive and maternal health indicators have not been measured in this setting but are necessary as part of an evaluation of a multi-ethnic pilot project exploring strategies to increase access to essential maternal health interventions. The goal of this study is to estimate coverage of maternal health services prior to this project and associations between exposure to human rights violations and access to such services.

**Methods and Findings:**

Selected communities in the Shan, Mon, Karen, and Karenni regions of eastern Burma that were accessible to community-based organizations operating from Thailand were surveyed to estimate coverage of reproductive, maternal, and family planning services, and to assess exposure to household-level human rights violations within the pilot-project target population. Two-stage cluster sampling surveys among ever-married women of reproductive age (15–45 y) documented access to essential antenatal care interventions, skilled attendance at birth, postnatal care, and family planning services. Mid-upper arm circumference, hemoglobin by color scale, and Plasmodium falciparum parasitemia by rapid diagnostic dipstick were measured. Exposure to human rights violations in the prior 12 mo was recorded. Between September 2006 and January 2007, 2,914 surveys were conducted. Eighty-eight percent of women reported a home delivery for their last pregnancy (within previous 5 y). Skilled attendance at birth (5.1%), any (39.3%) or ≥ 4 (16.7%) antenatal visits, use of an insecticide-treated bed net (21.6%), and receipt of iron supplements (11.8%) were low. At the time of the survey, more than 60% of women had hemoglobin level estimates ≤ 11.0 g/dl and 7.2% were Pf positive. Unmet need for contraceptives exceeded 60%. Violations of rights were widely reported: 32.1% of Karenni households reported forced labor and 10% of Karen households had been forced to move. Among Karen households, odds of anemia were 1.51 (95% confidence interval [CI] 0.95–2.40) times higher among women reporting forced displacement, and 7.47 (95% CI 2.21–25.3) higher among those exposed to food security violations. The odds of receiving no antenatal care services were 5.94 (95% CI 2.23–15.8) times higher among those forcibly displaced.

**Conclusions:**

Coverage of basic maternal health interventions is woefully inadequate in these selected populations and substantially lower than even the national estimates for Burma, among the lowest in the region. Considerable political, financial, and human resources are necessary to improve access to maternal health care in these communities.

## Abstract 2

Luke Mullany and colleagues examine access to essential maternal health interventions and human rights violations within vulnerable communities in eastern Burma.

## Editors' Summary

**Background.:**

After decades of military rule, Burma has one of the world's worst health-care systems and high levels of ill health. For example, maternal mortality (deaths among women from pregnancy-related causes) is around 360 per 100,000 live births in Burma, whereas in neighboring Thailand it is only 44 per 100,000 live births. Maternal health is even worse in the Shan, Karenni, Karen and Mon states in eastern Burma where ethnic conflicts and enforced village relocations have internally displaced more than half a million people. Here, maternal mortality is thought to be about 1000 per 100, 000 live births. In an effort to improve access to life-saving maternal health interventions in these states, Burmese community-based health organizations, the Johns Hopkins Center for Public Health and Human Rights and the Global Health Access Program in the USA, and the Mae Tao Clinic (a health-worker training center in Thailand) recently set up the Mobile Obstetric Maternal Health Workers (MOM) Project. In this pilot project, local health workers from 12 communities in eastern Burma received training in antenatal care, emergency obstetrics (the care of women during childbirth), blood transfusion, and family planning at the Mae Tao Clinic. Back in Burma, these maternal health workers trained additional local health workers and traditional birth attendants. All these individuals now provide maternal health care to their communities.

**Why Was This Study Done?:**

The effectiveness of the MOM project can only be evaluated if accurate baseline information on women's access to maternal health-care services is available. This information is also needed to ensure the wise use of scarce health-care resources. However, very little is known about reproductive and maternal health in eastern Burma. In this study, the researchers analyze the information on women's access to reproductive and maternal health-care services that was collected during the initial field implementation stage of the MOM project. In addition, they analyze whether exposure to enforced village relocations and other human rights violations affect access to maternal health-care services.

**What Did the Researchers Do and Find?:**

Trained survey workers asked nearly 3000 ever-married women of reproductive age in the selected communities about their access to antenatal and postnatal care, skilled birth attendants, and family planning. They measured each woman's mid-upper arm circumference (an indicator of nutritional status) and tested them for anemia (iron deficiency) and infection with malaria parasites (a common cause of anemia in tropical countries). Finally, they asked the women about any recent violations of their human rights such as forced labour or relocation. Nearly 90% of the women reported a home delivery for their last baby. A skilled attendant was present at only one in 20 births and only one in three women had any antenatal care. One third of the women received postnatal care and only a third said they had access to effective contraceptives. Few women had received iron supplements or had used insecticide-treated bednets to avoid malaria-carrying mosquitos. Consequently, more than half the women were anemic and 7.2% were infected with malaria parasites. Many women also showed signs of poor nutrition. Finally, human rights violations were widely reported by the women. In Karen, the region containing most of the study communities, forced relocation tripled the risk of women developing anemia and greatly decreased their chances of receiving any antenatal care.

**What Do These Findings Mean?:**

These findings show that access to maternal health-care interventions is extremely limited and that poor nutrition, anemia, and malaria, all of which increase the risk of pregnancy complications, are widespread in the communities in the MOM project. Because these communities had some basic health services and access to training in Thailand before the project started, these results probably underestimate the lack of access to maternal health-care services in eastern Burma. Nevertheless, it is clear that considerable political, financial, and human resources will be needed to improve maternal health in this region. Finally, the findings also reveal a link between human rights violations and reduced access to maternal health-care services. Thus, the scale of human rights violations will need to be considered when evaluating programs designed to improve maternal health in Burma and in other places where there is ongoing conflict.

**Additional Information.:**

Please access these Web sites via the online version of this summary at http://dx.doi.org/10.1371/journal.pmed.0050242.

- This research article is further discussed in a *PLoS Medicine* Perspective by Macaya Douoguih
- The World Health Organization provides information on all aspects of health in Burma (in several languages)
- The Mae Tao Clinic also provides general information about Burma and its health services
- More information about the MOM project  is available in a previous publication by the researchers
- The Burma Campaign UK and Human Rights Watch both provide detailed information about human rights violations in Burma
- The United Nations Population Fund provides information about safe motherhood and ongoing efforts to save mothers' lives around the world

## Introduction

Decades of oppressive policies, low-intensity conflict, and human rights violations in eastern Burma have forced hundreds of thousands of Burmese ethnic nationalities to flee into neighboring Thailand as refugees and/or economic migrants. Approximately 560,000 individuals are internally displaced within Shan, Karenni, Karen, and Mon States along Burma's eastern border [1]. With one of the world's least-functioning health systems [2], national health indicators in Burma (under-five mortality: 104/1,000) are among the worst in Southeast Asia [3]. In conflict-affected regions of eastern Burma, population-based household surveys indicate that the risks of infant (89 per 1,000 live births) and child mortality (218 per 1,000 live births) are substantially higher [4–6] partially due to widespread exposure to gross human rights violations [5].

While international humanitarian or relief efforts in this setting have been limited and subject to severe restriction from the military junta [7], numerous community-based organizations (CBOs) have been collecting population-based health information and implementing a range of public health programs among vulnerable communities despite attacks from the military regime (State Peace and Development Council [SPDC]) and active efforts to suppress or constrain these activities. For these groups, collecting health information in this setting is crucial for (1) understanding the needs of their target population so scarce resources can be appropriately targeted, (2) monitoring the progress of specific program implementation in this setting with its unique operational and logistical constraints and challenges, and (3) providing for the international community a more complete picture of the humanitarian needs and burden of disease than is possible through national statistics provided by the military regime.

Recently there has been increased recognition of the impact that conflict has on women's reproductive health outcomes and of the need for specific interventions to address these vulnerabilities [8,9]. The Back Pack Health Worker Team, a multi-ethnic organization that provides health services to internally displaced persons in active conflict zones of Karen, Karenni, Mon, and Shan states, has estimated the maternal mortality rate at approximately 1,000 per 100,000 live births, using pictorial vital event data collected by traditional birth attendants (TBAs) [6]; population-based surveys provide some supporting evidence [4], but collection of further data are warranted. While there is substantial uncertainty surrounding these mortality estimates, documented levels of underlying nutritional deficiency and disease among the internally displaced and refugee populations in this setting [1,10,11] heightens the risk of severe complications during pregnancy and increases mortality risk. Information on maternal health services within this population is generally limited to refugees within Thailand [12], and the degree to which ongoing exposure to human rights violations are related to access to such services within IDP communities has not been systematically explored.

In response to these maternal health needs, the Mobile Obstetric Maternal Health Workers (MOM) Project has developed a community-based network of providers, led by maternal health workers trained in basic emergency obstetric care, blood transfusion, antenatal and postnatal care, and family planning services. The overall goal of the project is to demonstrate the feasibility of increasing community-based coverage of essential maternal health services in under-served and vulnerable communities; details of the project have been previously published [13].

Characterizing health care access in these communities is complex. Although the SPDC has effectively blocked most forms of governmental and international humanitarian assistance, communities near the border in Thailand are sometimes able to access Thai health services, depending on proximity and the border security situation. Conversely, community members near the fringes of SPDC control are sometimes able to access SPDC services where they exist. In addition, both the human rights violations and the provision of health services by CBOs are strongly influenced by variances in the local security situation. Some ethnic groups continue active resistance, while others have signed cease-fires or have completely surrendered. Although the CBOs are based in the communities they serve, administration and supplies lines operate in a “cross-border” manner from the relative safety of neighboring Thailand, and thus proximity to Thailand increases access. CBO health providers are often mobile (“backpack” teams) or semi-mobile clinics that are able to relocate quickly in case of attack [14]. The communities selected for participation in the MOM project represent a slightly more stable subset of the eastern Burma conflict zones, with better access to the Thai border and the presence of semi-mobile clinics and other CBO health programs.

**Figure 1:**
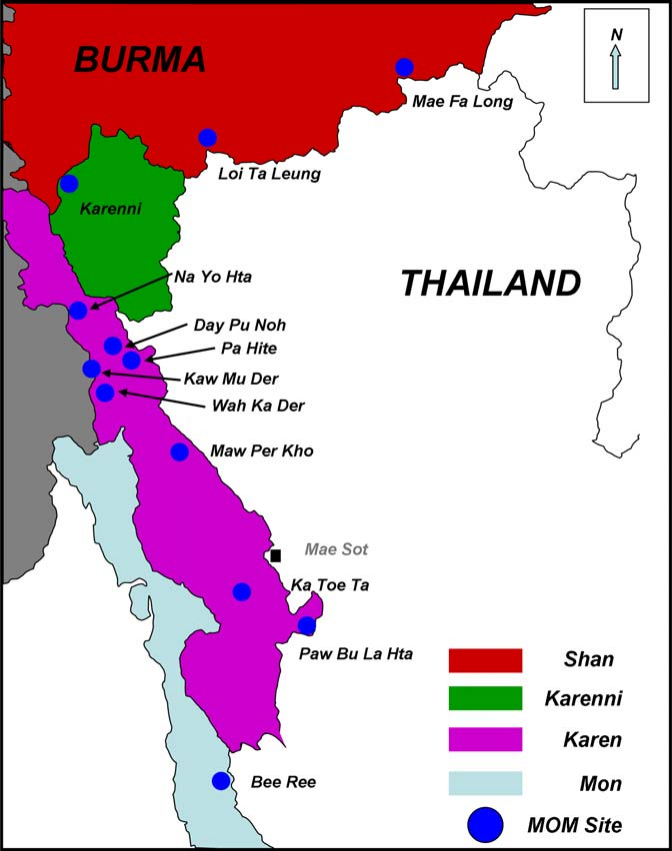
Location of Communities from Shan, Karenni, Karen, and Mon States Selected for Participation in the MOM Project and Included in the Reproductive Health Survey

At the initial field implementation stage of the MOM pilot project in 12 communities of Karen (*n* = 8), Karenni (*n* = 1), Mon (*n* = 1), and Shan (*n* = 2) states (Figure 1), project workers conducted a baseline assessment of coverage of reproductive, maternal, and family planning services, and experience of household-level human rights violations within the target population. Such data regarding access to essential services from populations within eastern Burma have been lacking. In this manuscript, the specific objectives are to (1) estimate coverage of maternal health services prior to the MOM project implementation, and (2) describe in a quantitative manner the associations between exposure to human rights violations and access to such services. These analyses are required to help guide the appropriate targeting of scarce resources toward reproductive health programs and illustrate the urgent needs among vulnerable populations in eastern Burma.

## Methods

This survey was conducted within the pilot areas of the MOM Project (Figure 1) between September 2006 and January 2007. The design, implementation, and general operational method followed that of previous population-based surveys conducted in this setting [4,5].

### Training of Surveyors

Survey workers (*n* = 22) were identified from the pilot communities, spoke the local language, and were known to their local community members. The 21-d training period included orientation to the instrument (questions, case-definitions, language-specific translations), interviewing techniques (probing, establishing rapport, anchoring techniques), role-playing, practice in sampling methods, and other procedures (informed consent, rapid diagnostic tests, measuring malnutrition using mid-upper arm circumference [MUAC], and assessment of hemoglobin using a color scale). During training, the leading trainer (CIL) recorded the MUAC of each of the workers; each worker then cross-measured each of the other survey workers, and the trainees' measures were compared with those of the leading trainer. Each survey team member was repeatedly trained on the MUAC procedure until 80% of his/her measures were within 0.5 cm of the leading trainer's assessments. For hemoglobin color scale and use of the rapid diagnostic test, competency was assessed qualitatively through direct observation during role-playing and pretesting. All survey workers received a small daily stipend during the periods of training and field activities.

### Survey Instrument

Background and demographic variables such as age, educational background, ethnicity, literacy, and occupation were collected, followed by a brief pregnancy and live birth history. Access to antenatal care (ANC) during the current or most recent pregnancy was examined, including coverage of malaria and anemia screening during pregnancy, iron/folate supplementation, antihelminthics, distribution of insecticide-treated nets, and overall number of antenatal visits and care providers (i.e., traditional birth attendant, medic, etc.). Place of delivery, type of delivery assistant, and access to postpartum care were recorded. A module on family planning and contraception was included to estimate unmet need and assist in directing of appropriate services during the project. Household composition and vital events (deaths, live births) in the prior 12 mo were also recorded.

The final module contained questions on exposure to human rights violations including forced labor, loss of food security, and forced displacement. These questions were previously developed by the Back Pack Health Worker Team to measure exposures with a demonstrated association with a range of health outcomes in eastern Burma [5], and may have an eventual explanatory role in the coverage and access of the MOM program. The variables relate directly to specific rights enshrined in international human rights law: forced labor (International Convention for Civil and Political Rights, Article 8 [15]), targeting of noncombatants (Geneva Convention IV, Article 3 & 27 [16]), theft and/or destruction of food supplies and other material goods essential for survival (Protocols Additional to the Geneva Conventions (II), Article 14 [17]), and forced displacement or relocation of civilian population (Protocol II, Article 17 [17]; Universal Declaration of Human Rights Article 13 [18]).

The survey instrument was translated into four languages (Burmese, Shan, Karen, and Mon) with repeated back-translation for standardization of content and meaning. All modules underwent three rounds of village-based pilot testing.

### Procedures

Workers explained the purpose and procedures of the survey, and read a verbal consent statement. Survey questions were then read to women agreeing to participate, and responses recorded on paper forms. In addition to the questions, the respondent's MUAC was estimated and hemoglobin levels were estimated using a simple hemoglobin color scale (Teaching Aids at Low Cost, UK) [19]. Women were also screened for malaria parasitemia (falciparum) using a rapid diagnostic test (Paracheck, Orchid Biomedical Systems, India). This rapid test has a higher documented sensitivity (85%–94%) and specificity (89%–99%) for asymptomatic infection than field microscopy [20–22]. Women testing positive for malaria parasitemia received a course of artemisinin combination therapy [23], unless pregnant. Pregnant women were referred to the local MOM project worker for treatment. All women with estimated hemoglobin levels < 8 g/dl were provided with 90 d of iron and folic acid and referred to their MOM project worker for follow up. Those with levels 8–11 g/dl were referred to a MOM project worker for treatment and educational messages.

### Sampling Procedure

All data were collected from communities participating in the MOM pilot project and are a population-based sample, representative of the participating communities, but not necessarily representative of the broader population in eastern Burma. In each of the 12 project communities (each of which includes a number of villages), census information (aggregated at the household level) was collected by local workers for each village within the target prior to conducting the survey. The total population size of all the villages within the participating MOM communities from Karen, Karenni, Shan, and Mon states is approximately 60,000. For the purposes of the survey, the eight communities in Karen state were grouped into four Karen survey areas based on geographical proximity and population size. Each of the communities in the Karenni, Shan, and Mon additionally contributed one survey area. To select survey respondents in of these areas, two sampling schemes were followed to reach the desired sample size in each area (see Sample Size, below); both schemes provide population-based representative samples within the area. In the four Karen and one Karenni areas, where each community has many separate small villages and households within villages are not generally organized systematically, a standard two-stage cluster sampling scheme [24,25] was followed. In the first stage, the area-specific list of villages and the corresponding aggregate census information at the village level was used to select, proportionate to population size, 40 village-based clusters in each of the five areas. In the second stage, ten households with at least one ever-married women of reproductive age (15–45 y) were selected using proximity sampling. In the Shan and Mon areas, where there are few villages and households within villages are organized in rows, simple systematic (interval) sampling [25] of households within each village was used.

### Sample Size

The size of the survey was determined after considering both logistical constraints unique to working in this population and the desired level of precision of the estimates of progress in increasing coverage throughout the MOM project. The original sample size calculation was based on the number of surveys needed to detect area-specific differences between the baseline and end-of-project estimates of one key indicator: the proportion of women with anemia (∼400 surveys per area). The sample size was 50% higher (i.e., 60 clusters of ten households, ∼600 surveys) for one of the Karen survey areas, as this site was originally planned as an independent non-MOM project site (this site did not collect information on malaria parasitemia, MUAC, or hemoglobin levels of respondents). As the survey design, cluster and household selection, survey instrument, and data management were identical in all other respects, this area with a slightly higher sample was also included in the total. Thus, the total sample size (*n* = 3,000) available for examining baseline access to maternal health interventions was fixed. Assuming design effect (due to cluster sampling) of 2.0, this size provided sufficient data to estimate coverage of any binary indicator (i.e., proportionate coverage of single interventions) in this cross-sectional survey with absolute precision of approximately 2.5% (overall) and 6.9% (within areas), respectively. The sample size was not selected with a priori intent to estimate any subsequent changes in mortality as a result of the MOM project; however, the size of the sample was sufficient to provide point estimates of child, infant, and neonatal mortality in the 12 mo prior to the survey.

### Analysis

Surveys were returned to the MOM office and entered into a secure, password-protected Microsoft Access database. Binary and categorical variables were summarized by proportions and tabulations. Continuous variables were summarized using the mean, standard deviation (SD), and range. Differences between groups were assessed using chi-square tests and/or logistic regression models with confidence intervals adjusted for the cluster-survey design. Coverage for antenatal care services during the last pregnancy was estimated among the subset of respondents who reported a pregnancy within the 5 y prior to the survey. Currently pregnant primiparous women and women who had never been pregnant or whose last pregnancy was more than 5 y ago were excluded. For those whose pregnancy resulted in a live birth, estimates of skilled attendance at birth, place of birth, postnatal care, maternal report of early initiation of breast-feeding (within 24 h of birth), and receipt of postpartum vitamin A as reported by respondents, were additionally recorded. In this survey, women with an unmet need for limiting or spacing pregnancy were defined as follows: (1) nonpregnant women who were not using a modern contraceptive method to delay conception and who did not want any more children or wanted to delay conception beyond 2 y, or (2) women who reported that they desired their current pregnancy to have been either avoided or delayed. Neonatal, infant, and child mortality rates were estimated for the overall pilot project population. Exposure of household members to human rights violations was estimated separately for each ethnic area; among Karen communities the association between rights violations and access to antenatal interventions and outcomes were modeled using logistic regression. The number of recently delivered (< 1 y) women were insufficient in the smaller samples from Shan, Mon, and Karenni regions to examine these relationships. Adjustment of variance estimates was necessary to account for the cluster-survey design; variance estimation was done using Taylor linearization, and all analyses were performed using Stata 9.0 (StataCorp, College Station, TX) and its associated “svy” suite of commands.

### Ethical Approval

The design and conduct of these surveys was approved by the Johns Hopkins Committee on Human Research and the MOM Monitoring & Evaluation Committee, an ad hoc, independent border-based committee charged with overseeing monitoring efforts.

## Results

### Sampling Frame and Coverage

The total estimated population in the pilot areas at the time of the survey was 59,042 (aggregate census data). Survey teams approached 2,914 of 3,000 (97.1%) planned households between May and December 2006. The participation rate among approached households was high; after 25 refusals (reason unspecified), 2,889 (99.1%) households contributed information for the analysis (Table 1). Excluding the single region where malaria parasitemia, MUAC, and hemoglobin levels were not included in the instrument, the number of approached (2,343/2,400; 97.6%) and participating households (2,340/2,343; 99.9%) was similarly high.

**Table 1:**
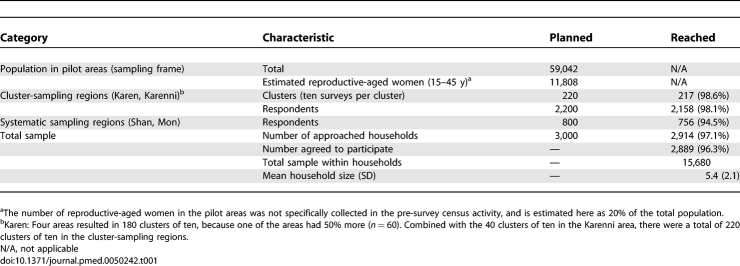
Survey Sample and Basic Demographic Characteristics of Participating Respondents

### Characteristics and Tested Health Indicators among Respondents

The distribution of the sample across ethnic groups reflects the selection of communities in the pilot program: 1,728 (59.8%) women were ethnic Karen, with Karenni (*n* = 399, 13.8%), Mon (*n* = 387, 13.4%), Shan (*n* = 290, 10.0%) and other (*n* = 80, 2.8%; Burman, Arakan, Lahu, Palaung, Pa O) ethnicities reported. The mean age (SD) of participating women was 30.6 (7.5) y (range: 15–45 y) with the majority reporting less than 5 y (*n* = 2,511, 87%) or no (*n* = 1,559, 54%) education. Shan and Mon respondents were predominantly Buddhist (> 98%), while Karenni were mainly Christian (83%), and Karen were a mix of Christian (44%), Buddhist (42%), and traditional (animist) religions (13%).

A total of 464 (16.9%) respondents reported that they were currently pregnant, with the highest proportion occurring among Karenni women (26.9%). Data on malaria parasitemia, MUAC, and hemoglobin levels of respondents were collected from, respectively, 98.2% (2,298/2,340), 98.3% (2,300/2,340), and 98.2% (2,297/2,340) of respondents in the regions where these procedures were included. At the time of the survey 7.4% (*n* = 171) of women were positive for falciparum malaria, and this differed between pregnant (*n* = 40, 10.4%) and nonpregnant (*n* = 117, 6.5%) women (OR = 1.67, 95% CI 1.11–2.51). Approximately 61.1% (*n* = 1,403/2,297) were estimated to have hemoglobin levels ≤ 11.0 g/dl; such levels were associated with malaria parasitemia (OR = 2.59 [1.70–3.95]) but were not significantly different between pregnant and nonpregnant women. The mean (SD) MUAC measure was 24.4 cm (2.5) and the interquartile range was 23.0–25.8 cm. The proportion of women with MUAC values below 22.5 [26] was 19.3% (*n* = 444/2,300).

### Pregnancy History and Estimates of Mortality

The mean age at marriage and first pregnancy was 20.8 y, and 11.8% (334/2,816) of women reported being married by the age of 16 y. Including those who were currently pregnant, 2,750 (95.1%) women reported ever being pregnant, and the mean (SD) number of pregnancies was 3.9 (2.6); range 0–15; women in the highest age group (40–45 y) on average reported 6.9 pregnancies. Among those reporting ever being pregnant, 2,609 (94.8%) had given birth to a live baby and the mean (SD) number of live births was 3.8 (2.3). The ratio of live births to population (46.5 per 1,000) was high, but overestimated the true crude birth rate because only households with women of reproductive age were sampled. More than one-third of respondents reported the death of one or more of their live-born children; this proportion differed between ethnic groups (*p* < 0.001) with the highest proportion reported among Karen (39.9%) and Shan (39.9%) and the lowest among the Mon (9.9%). In these selected pilot communities, the child mortality indicators were moderate to high: there were 636 live births, 15 neonatal deaths (23.6 per 1,000 live births [95% CI 10.2–37.0]), 39 infant deaths (61.4 per 1,000 live births [95% CI 36.8–86.9]), and 80 child (< 5 y) deaths (125.9 per 1,000 live births [95% CI 92.1–160.0]) reported.

### Antenatal Care

Among women who reported ever being pregnant, there were 2,252 (81.9%) women for whom the last pregnancy concluded in the previous 5 y. ANC was provided in the last pregnancy to 885 (39.3%) respondents; any care during pregnancy and receipt of four or more visits was more common among Mon and Shan communities than Karen and Karenni (*p* < 0.001). ANC was generally provided in clinics run by CBOs or in the home by TBAs. Coverage of basic ANC interventions varied by area, but was generally low, especially in the Karen area (Table 2; all interventions varied significantly by area). Overall coverage rates were also low for basic interventions such as full (at least two doses) coverage of tetanus toxoid (14.3%), antihelminthic during pregnancy (4.1%), and provision of 3 mo or more of iron and folic acid (11.8%). All areas are endemic malaria regions, yet only one-fifth (21.6%) of women utilized an insecticide-treated net during pregnancy and only 9.8% were tested for malaria. In general, women in Mon areas had increased access to essential ANC interventions.

**Table 2:**
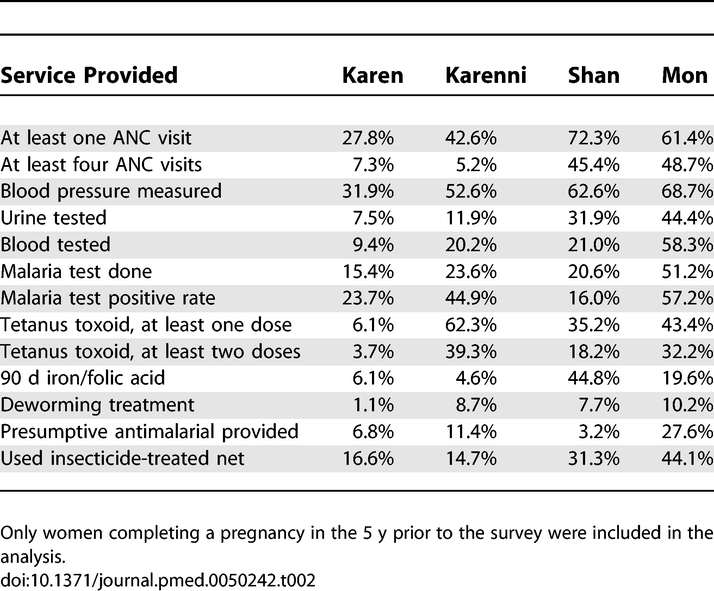
Antenatal Care Services in Last Reported Pregnancy by Area

### Labor, Delivery, and Postnatal Care

Among the 2,252 women concluding a pregnancy within the previous 5 y, there were 2,104 (93.4%) women who reported a live birth, and information on labor and delivery and postnatal care was collected among these women. The majority delivered at home (87.6%) or in one of the CBO clinics in their area (6.4%). Only 3.4% of women reported delivering their baby in a Burmese (1.7%, mostly Karenni, Shan, or Mon women) or Thai hospital (2.7%, mostly Shan women). Skilled attendance at birth was defined as a doctor or nurse/midwife; coverage was extremely low in Karen (1.9%) and Karenni (5.1%) areas and overall (5.1%). Deliveries attended by skilled attendants were concentrated in Mon (13.1%) and Shan (13.6%) areas where some women had opportunity to travel across the border to Thailand to access hospitals. Overall, the primary attendant was most commonly reported as a traditional birth attendant (TBA) (61.9%), family member/relative (22.9%), or local health worker from the CBO clinic in the area (9.6%).

Postnatal care (PNC) was provided within 1 wk of delivery for 33.7% of respondents. Among Karen women (27.4% received PNC) this postnatal contact was normally made in the home (93.4%). Compared with Karen women, Shan and Mon women reported PNC at a greater rate (63.4% and 49.3% of women, respectively). When PNC was sought, these Shan and Mon respondents were more likely than Karen or Karenni women to have access to PNC in a clinic or hospital (60.9% versus 13.9%, OR = 8.38 [95% CI 4.12–17.03]), reflecting the greater access, especially among Shan women, to facilities across the border in Thailand. Receipt of postpartum vitamin A was low overall (12.3%) and especially among Karen women (4.3%). Early initiation of breastfeeding (within the first 24 h) was high overall (93.7%) and within each area, and feeding of colostrum exceeded 80% in all areas except among Shan women (68.2%). Exclusive breastfeeding through 6 mo was less common, however. Overall, 16.5% of women reported exclusively breastfeeding for 6 mo; most of these were in Shan and Karen areas where approximately one in five women reported the practice.

### Family Planning

Information on family planning was available for 2,861 (99.0%) of the sample. About one quarter (*n* = 725, 25.3%) of all responding women reported doing anything to delay pregnancy. Among those employing any contraceptive method, modern methods were generally used (*n* = 685, 94.5%), with only small numbers of women reporting exclusive breastfeeding, abstinence, withdrawal, or calendar-based methods. The two most common methods both overall and within each site were depot medroxy-progesterone acetate (Depo-Provera) (73%) followed by oral contraceptive pills (20.9%). Unmet need in this population was high (*n* = 1,764, 61.7%) and the greatest unmet need was observed among Karen (74.8%) and Karenni (70.1%) communities and the lowest in the Shan (37.5%) and Mon (17.2%) communities (*p* < 0.001).

### Human Rights Violations

Information regarding household experience of human rights violation is shown in Table 3. Overall estimates are not provided, given the considerable variation between areas. Reports of violations were uniformly low in the Mon area (one respondent reported forced labor). In the Karenni ceasefire region, however, the most common rights violation was forced labor; almost one-third of households in this area (*n* = 128, 32.1%) reported one or more individuals being forced to work. Among the Karen communities, which are non-ceasefire areas and are more likely to experience sporadic, active conflict, reports of forced labor were substantially lower (1.5%). More than 10% (*n* = 180, 10.5%) of Karen households, however, reported being forced to move in the previous 12 mo; most of these were concentrated in northern regions of Karen State. In the Shan area, all rights violations included in the instrument were reported at high rates; threats to food security, forced relocation, forced labor, and direct attacks were all reported by one-fifth to one-quarter of the responding households.

**Table 3:**
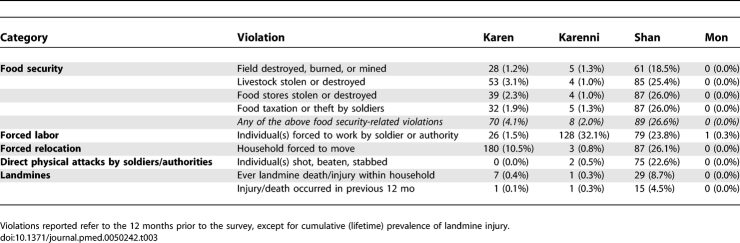
Number and Prevalence of Human Rights Violations within the 12 Months Prior to Survey, by Area

The odds of anemia, positive parasitemia, low MUAC, unmet need, access to ANC interventions (individually and combined), access to a postnatal care visit, and skilled attendance at delivery were compared between Karen households that were or were not exposed to forced movement or food security violations in the 12 mo prior to the survey (Table 4). Odds of hemoglobin ≤ 11 g/dl were higher among women in households experiencing either forced displacement (OR = 1.51 [95% CI 0.95–2.40]) or security-related loss of food (OR = 7.43 [2.21–25.3]). While receipt of antenatal interventions was low overall, access was substantially lower among households reporting forced displacement; odds of having access to none of the core interventions in the last pregnancy was almost six times higher (OR = 5.94 [2.23–15.8]) among those forced to move. This trend in access to interventions was also seen among those reporting food security violations, but statistical power was low.

**Table 4:**
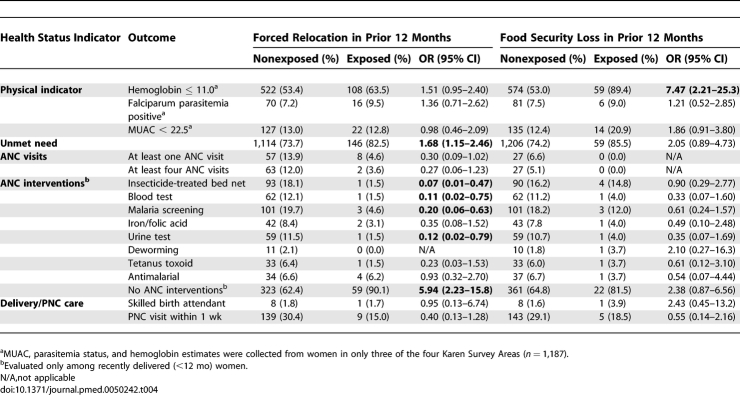
Association between Selected Maternal Health Indicators and Access to Antenatal Interventions and Human Rights Violations among Karen Households

## Discussion

In these selected populations in eastern Burma, access to essential maternal health interventions during pregnancy is generally low. Importantly, assistance at delivery by individuals who can provide skilled services—especially basic components of obstetric care—saves maternal lives but is universally rare; assistants are normally TBAs or neighbors and friends. More than 7% of ever-married reproductive aged women were positive for falciparum malaria, and parasitemia rates were significantly higher among pregnant women. Finally, there is substantial unmet need for modern contraceptives in all four of the areas.

The indicators and coverage estimates provided here are strikingly worse than the already low national estimates for Burma that have been provided by various institutional reports. These include coverage of four or more ANC visits as recommended by the World Health Organization (76% [27]), presence of a skilled attendant at birth (57% [28]), use of modern method of contraception (33% [29]), and unmet need (46% [30]). While methodological differences exist among the sources cited above and between those and the current survey, such differences are unlikely to explain the lower coverage in this setting. In fact, a basic comparison of the data presented here with the above indicators from a national health system in crisis underestimates the severity of the problem in eastern Burma. The extent of the disparity in access to basic maternal health services is better highlighted when one considers the coverage of ANC (86%), skilled birth attendants (99%), and modern contraceptive use (70%) in Thailand [31]. Specific health indicators measured in this survey, including Plasmodium falciparum positivity rates, hemoglobin estimates, and MUAC measures are also poor. The prevalence of P. falciparum is consistent with previous estimates from population-based surveys in both these settings in eastern Burma [5,11] and among refugee and migrant populations in eastern Thailand [32,33]

The communities included in this survey were selected for participation in the MOM project on the basis of some already existing CBO health services and access of health workers to training in Thailand. Thus, the baseline estimates and outcomes are not necessarily representative of the wider population in Shan, Karen, Karenni, or Mon states. Generalizability of results is especially cautioned in Shan, Mon, and Karenni regions, where estimates come from a single target area within the more broadly defined state. Further information from other communities in these areas is required to fully characterize the levels of access to interventions measured here. It is unlikely that access to services in communities not represented here would be higher. Rather, the estimates given here may overestimate the level of services in the overall population, which is more directly affected by conflict and exposed to ongoing human rights violations. This is especially true for the Shan area where the pilot sites are closer to the border of Thailand (Figure 1) and at least a small subset of the population has been able to receive limited services by crossing into Thailand. This is generally not the case in the selected Karen populations, and, on average, the coverage indicators for Karen communities were substantially lower than in the other areas. Even these estimates for Karen women, however, may overestimate the level of access in the wider Karen region, because these communities were selected for participation into the MOM project on the basis of minimal level of security to allow workers to travel to Thailand for training. In addition, the selected areas had basic clinics and health programs (such as child vitamin A distribution and malaria control/bed-net programs) staffed by health workers from CBOs [13]. Reflecting this selection, vital indicators like under-five and infant mortality rates in these selected communities are lower than previously reported from areas within the same eastern states of Burma where there is more displacement and more active conflict [4–6].

The association between anemia and forced displacement (50% greater) and food insecurity (greater than 7-fold) is remarkable. Although this association does not prove causality, the association between these violations and the lack of ANC services that would reduce anemia is compelling, especially forced displacement (nearly 6-fold). There was a trend toward a lower likelihood of receiving individual ANC interventions among those exposed to forced displacement and decreased food security in the Karen communities. These associations in no way imply causality (there are no temporal data) and are limited by a range of potential biases and unmeasured confounders. The data do suggest, however, that exposure to rights violations might be an important cofactor in the likelihood of future access to the MOM project interventions, and that the degree to which these factors interact with access to public health programs might be quantified. It is important to note that there were some inconsistencies in the relationship between health interventions and human rights violations. For example, the likelihood of having a skilled birth attendant or having MUAC < 22.5 cm did not differ by forced relocation status, and skilled birth attendance was higher among women exposed to food security violations (although this was not statistically significant).

The potential impact of ongoing instability in the Karen communities can also be seen from an ecological perspective. In Mon and Karenni regions where cease-fire agreements have been reached, there are either very low reports of human rights violations (Mon) or violations are restricted to certain types (forced labor in Karenni region). At the same time, basic intervention coverage estimates appear substantially higher in the Mon region, although these levels are still inadequate compared to national figures from Burma and neighboring Thailand. In Karen communities, where conflict has affected communities for decades and has escalated dramatically since the military regime's move of the capital city to Pyinmana (Nyapidaw) in 2005, there is practically no functioning public health sector, and indicators of structural services (ANC, PNC, skilled attendance at delivery) were lowest among the four communities studied. As noted above with the direct approach, there are some inconsistencies in the ecological data; for example, while reports of violations were generally higher among Shan communities, access to maternal health care was also generally higher among these women (especially ANC visits and some associated interventions). Such inconsistencies highlight both the importance whenever possible for a direct, rather than ecological, approach and the need for further work in this area of quantifying associations between human rights violations and health outcomes.

The MOM project is specifically working to improve access and use of maternal health services, and not directly working to decrease these rights violations. The innovative community-based delivery model of the MOM project, which focuses on mobility of services, might enable improvement in access despite these ongoing violations. However, these data suggest that here and in other similar settings where rights violations are ongoing, the prevalence of such exposures and an accounting of their potential impact on the success/failure of the program must be carefully considered during the evaluation stage.

Increasing access to antenatal, labor and delivery, and newborn care services in eastern Burma is essential in order to improve the overall health status of these vulnerable populations. These data illustrate the magnitude of the need and serve as a call to action to include an emphasis on maternal and more comprehensively, reproductive health services in health programs targeting these communities. Specifically, an emphasis on increasing family planning services and providing essential and focused ANC interventions such as malaria screening and treatment in pregnancy, iron and folic acid supplementation, antihelminthic treatment, and appropriate counseling in home-based essential newborn care. Reductions in maternal mortality and morbidity require assistance at delivery by individuals trained to provide at least the basic components of emergency obstetric care. In the pilot communities focused upon in this study, health care leaders of the Shan, Karen, Karenni, and Mon states are currently collaborating through the MOM pilot project to provide these services using a multitiered layer of community-based maternal health workers, health workers, and traditional birth attendants (to be completed in 2009). While that program aims to provides valuable insight into alternative strategies for delivering these services in such settings, substantially greater efforts and resources, including political, human, and financial, will be necessary to scale up activities and reach the hundreds of communities in need.

## Abbreviations

ANC: antenatal care
CBO: community-based organization
CI: confidence interval
MOM: Mobile Obstetric Maternal Health Worker
MUAC: mid-upper arm circumference
PNC: postnatal care
SD: standard deviation
SPDC: State Peace and Development Council
TBA: traditional birth attendant
